# Immunotherapy using Histobulin™ in psoriasis: A case report

**DOI:** 10.1002/ccr3.5831

**Published:** 2022-05-12

**Authors:** Hyuk Soon Kim, Geunwoong Noh

**Affiliations:** ^1^ Department of Biomedical Sciences College of Natural Science and Department of Health Sciences The Graduate School of Dong‐A University Busan Korea; ^2^ 464474 Allergy and Clinical Immunology Center Cheju Halla General Hospital Jeju‐si Korea

**Keywords:** biologics, Histobulin™, immunoglobulin/histamine complex, immunotherapy, psoriasis

## Abstract

There is no cure for psoriasis. A psoriasis patient was treated with Histobulin™. The patient's clinical symptoms and signs disappeared after the eighth injection and did not recur for more than 18 months. Histobulin™ was effective in the treatment of psoriasis and is suggested as a curative therapeutic for psoriasis.

## Abstract 2

There is no cure for psoriasis, and early treatment is recommended to improve skin manifestations and systemic inflammation, which can lead to comorbidities in various organs. Histobulin™ was effective and induced remission in a psoriasis patient.

## 1 INTRODUCTION

Psoriasis is a chronic immune‐mediated inflammatory disease that affects 2% to 4% of the population worldwide.^1^ Psoriasis is an immune‐mediated disease, and a complete cure is difficult to achieve as patients experience progressive recurrences.^2^ Psoriasis is a relapsing and remitting condition that may be exacerbated by environmental factors such as trauma, stress, and infection.^3^

There is no cure for psoriasis, but several treatment options exist,^4^ including topical corticosteroids, retinoids, coal tar preparations, dithranol, salicylic acid, and vitamin D analogs; phototherapy with ultraviolet (UV) B or UVA plus psoralen; and systemic immunosuppressants, such as oral corticosteroids, methotrexate, cyclosporin, and acitretin. Hydroxyurea, sulfasalazine, and tacrolimus have also been used in patients who fail to respond to more conventional therapies. The immunopathogenesis of psoriasis is now well understood, and biologics have been developed and trialed recently with good effects. Most importantly, psoriasis is understood as a systemic disease. To prevent systemic comorbidities, early systemic treatment with biologics, including etanercept, adalimumab, certolizumab infliximab, certolizumab, and ustekinumab, is recommended due to the centrality of their targets in disease pathogenesis.^5^

Histobulin™ (Green Cross PD, Korea) is a histamine‐fixed immunoglobulin preparation comprising 0.15 μg of histamine dihydrochloride and 12 mg of immunoglobulin.^6^ This preparation was developed for the regulation of blood serum levels by histaminopexic effects^7^ and has been shown to be effective in treating patients with allergic rhinitis, bronchial asthma, chronic urticaria, and atopic dermatitis (AD).^8^, ^9^, ^10^, ^11^ This case report describes the treatment of a patient with psoriasis with Histobulin™ therapy, and complete remission of psoriasis was observed. Therefore, Histobulin™ is suggested as a curative therapeutic.

## 2 CASE REPORT

A 15‐year‐old Korean male patient visited the Department of Allergy and Clinical Immunology, Cheju Halla General Hospital, due to allergic rhinitis for several years and the presence of round and scaly skin eruptions on the whole body for 2 months. He had no specific family history or past medical history. The patient felt slight itching on the skin lesion sites. Concerning allergic rhinitis, he had suffered from frequent rhinorrhea, sneezing, nasal congestion, and itching of the nose for several years as typical clinical manifestations of allergic rhinitis. The patient had taken 5 mg of levocetirizine once a day for the relief of allergic rhinitis symptoms when his daily life was affected. The development and progression of allergic rhinitis and psoriasis were not found to be related to each other in the medical history or clinical progression.

Basic allergy tests (blood tests and a skin prick test) were conducted. He underwent blood tests for a complete blood count (CBC) with differential serum eosinophil cationic protein and serum total IgE levels. Specific IgE levels for the allergens were found using a multiple allergosorbent test (MAST, Green Cross PD, Korea). In the MAST, the specific IgE levels for 41 allergens were evaluated as described in a previous report (see the Supplementary material).^11^ The test results showed the specific IgE level for each allergen, and a normal negative range was between 0.000 and 0.349 IU/ml.

A skin prick test was also performed for 53 allergens as described in a previous report (see the Supplementary material).^11^ Histamine hydrochloride 10 mg/ml was used as the positive control, and physiological saline was used as the negative control. The wheal size was measured. Reactions were read after 15 min and described as negative (0, no reaction), 1+ (reaction greater than a control reaction but smaller than half the size of the histamine wheal), 2+ (equal to or more than half the size of the histamine wheal), 3+ (equal to or more than the size of the histamine wheal), and 4+ (equal to or more than twice the size of the histamine wheal). The minimum size of a positive reaction was 3 mm.

The severity score was evaluated using the Psoriasis Area and Severity Index (PASI).^12^ Four body regions were assessed according to erythema, infiltration, desquamation, and body surface area involvement. The degree of severity (per body region) was scored from 0 to 4. The surface involvement (per body region) was scored from 0 to 6. The PASI produces a numeric score ranging from 0 to 72. Skin biopsy was performed to confirm the diagnosis of psoriasis.

The patient underwent laboratory tests, a skin prick test, and PASI scoring before and after treatment. White blood cell (WBC) counts were normal at 5.57 before treatment and 7.99 after treatment (normal range: 3.9–11.0 1000/μl). In the differential counts of WBCs, the neutrophil, lymphocyte, eosinophil, and basophil fractions were within the normal range. Blood eosinophil cationic protein levels were as high as 37.9 before treatment and decreased to 35.5 after treatment (normal range: 0–24 ng/ml). After Histobulin™ therapy, the serum IgA level was evaluated for selective IgA deficiency and was normal at 95.7 (normal range 70–400 mg/dl). The total IgE level was normal at 203 before treatment and 297 after treatment (normal range: less than or equal to 350 IU/ml).

In the MAST, the specific IgE levels for *Dermatophagoides pteronyssinus* (Dp), *Dermatophagoides farina* (Df), cats, shrimp, and timothy grass were positive before treatment and decreased after treatment for all items (Table 1). In the skin prick test, the changes in reactions according to the allergens were variable and insignificant.

**TABLE 1:** Sensitization profiles to exogenous allergens by a multiple allergosorbent test (MAST, Green Cross PD, Korea) and a skin prick test (SPT)

MAST (Normal Range 0.35 IU/ml>)			SPT (Grade)		
Allergens	Before Tx	After Tx	Allergens	Before Tx	After Tx
Dp	6.17	2.95	Dp	0	4+
Df	3.35	2.53	Df	0	3+
Cat	1.26	1.02	Cat	0	3+
Timothy grass	0.57	0.39	Timothy grass	3+	3+
Shrimp	0.63	0.00			
			Grass mix	4+	4+
			Orchard	4+	3+
			English rye grass	0	4+
			Japanese cedar	0	3+

Note

For the MAST, the test results show the level of the specific IgE for each allergen, and a normal negative range is between 0.000 and 0.349 IU/ml. The SPT results are described as negative (0, no reaction), 1+ (reaction greater than a control reaction but smaller than half the size of the histamine wheal), 2+ (equal to or more than half the size of the histamine wheal), 3+ (equal to or more than the size of the histamine wheal), and 4+ (equal to or more than twice the size of the histamine wheal). The minimum size of a positive reaction is 3 mm.

**FIGURE 1:**
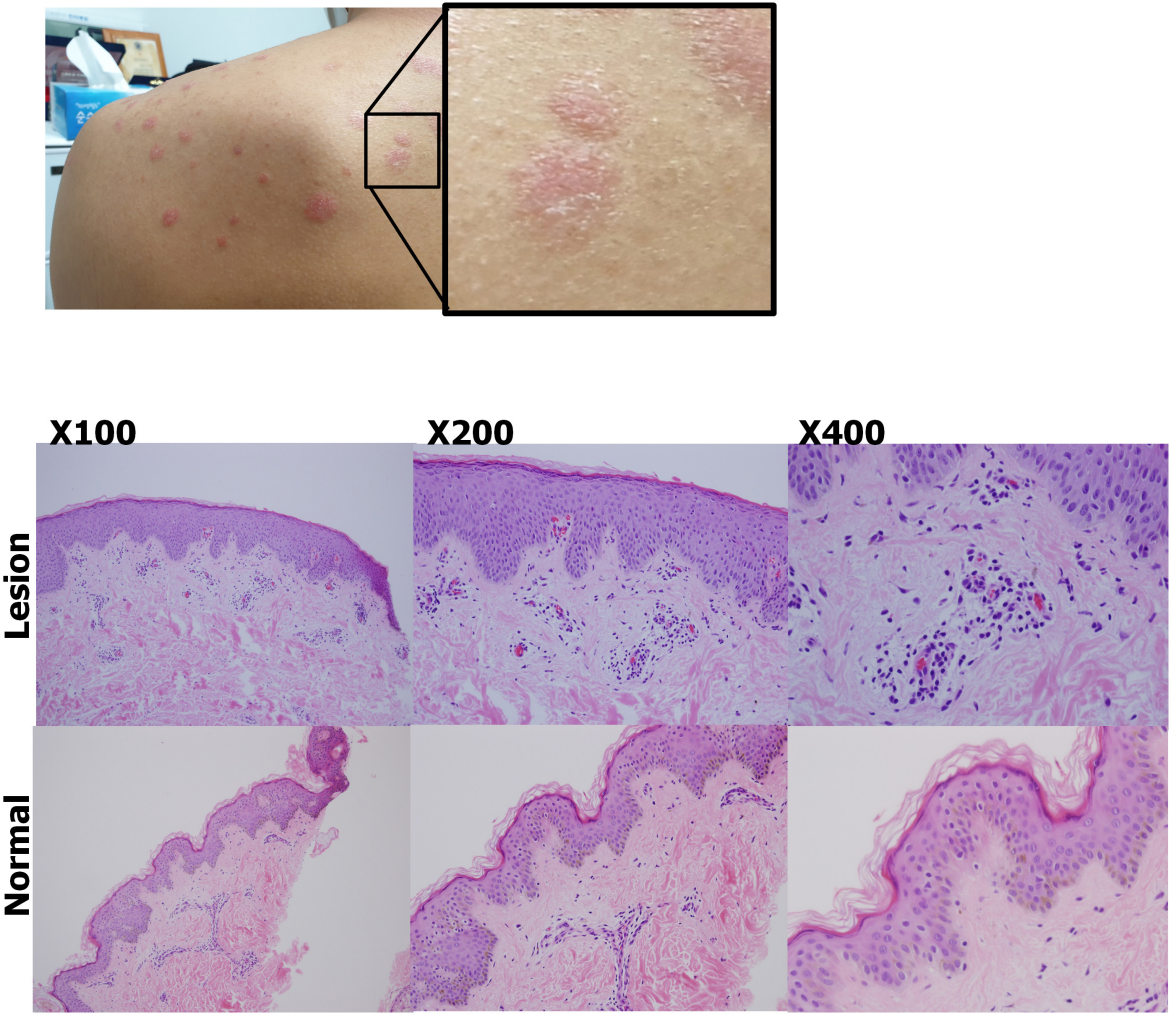
Photographs and pathological findings. The patient showed scaly round skin eruptions on the whole body, which is typical for psoriasis. The pathological findings of the lesions were suggestive of subacute spongiotic dermatitis. The lesions showed acanthosis, a microscopic focus of spongiosis with overlying microscopic parakeratosis and the absence of keratohyalin granules. Acanthosis with elongated epidermal ridges was observed (HE X 100). Club‐shaped epidermal ridges (HE X 200) and elongated dermal papillae containing dilated capillaries (HE X 400), which are typical of psoriasis, were observed. The pathological diagnosis was psoriasis

Skin biopsy was performed at the lesion site and an unaffected site on the back. The specimens were 0.4 × 0.3 × 0.5 cm. HE staining was performed. The pathological finding of the lesion site was suggestive of subacute spongiotic dermatitis. The results showed acanthosis, a microscopic focus of spongiosis with overlying microscopic parakeratosis and the absence of keratohyalin granules. Acanthosis with elongated epidermal ridges was observed (HE X100). Club‐shaped epidermal ridges (HE X 200) and elongated dermal papillae containing dilated capillaries (HE X 400), which are typical of psoriasis, were observed. The pathological diagnosis was psoriasis (Figure 1).

**FIGURE 2:**
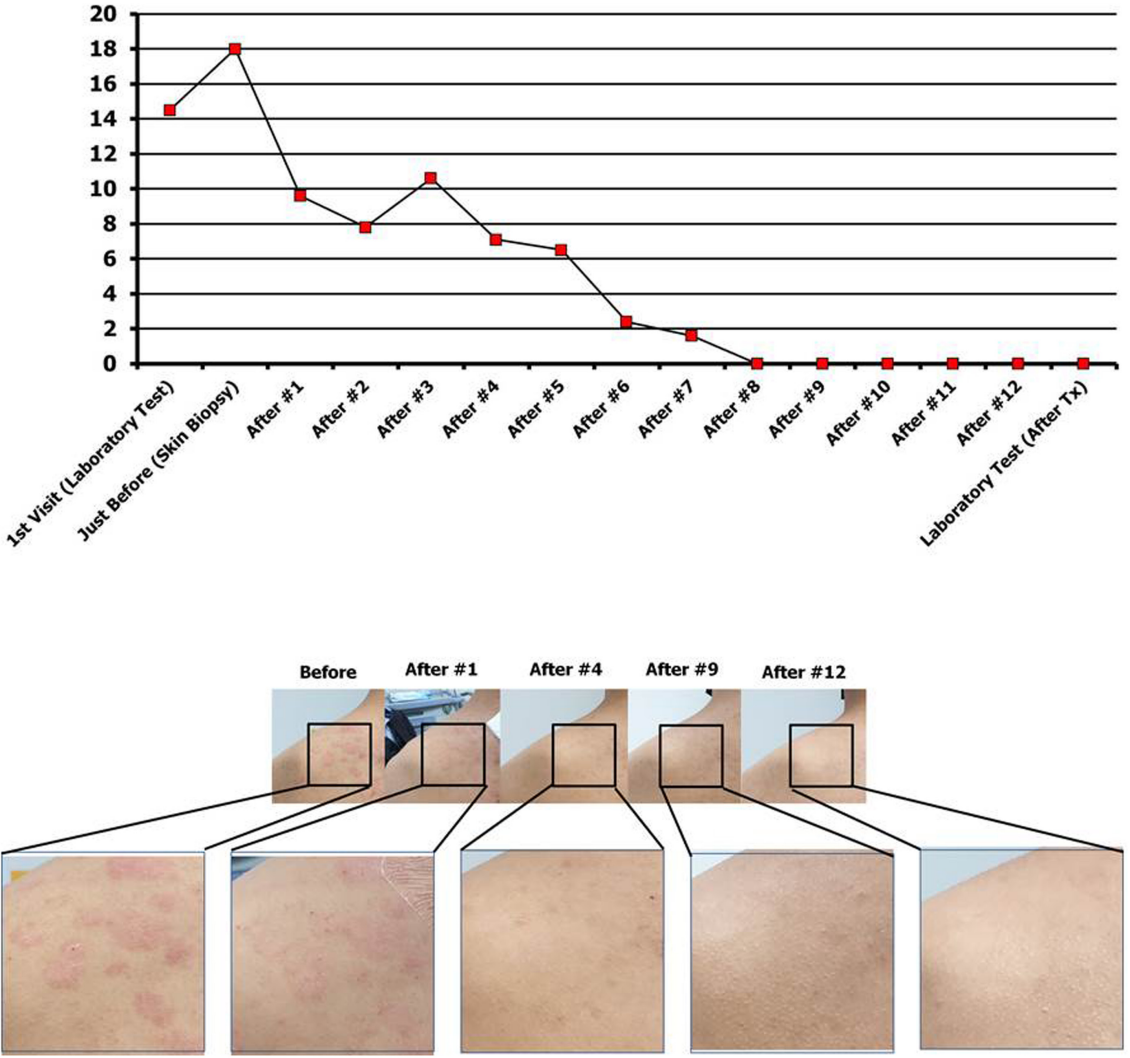
Clinical progression of Histobulin™ therapy in a psoriasis patient. The patient's psoriasis progressed, and the PASI score increased from 14.5 to 18 points over 2 weeks, during which time laboratory tests and skin biopsy were performed. The clinical response was rapid, and the patient's symptoms and signs improved after the first injection of Histobulin™. Although the patient temporarily showed some irritation after the third injection, the clinical manifestations, including skin lesions, improved continually and completely disappeared after the eighth injection. The patient showed no symptoms or signs of psoriasis for 4 weeks, during which 4 subsequent injections were administered (from the ninth to the twelfth injection). The patient stopped taking medication, and the patient did not experience recurrence for more than 6 months

The final diagnosis was allergic rhinitis and psoriasis. Histobulin™ therapy for allergic rhinitis was initiated, and the clinical severity of psoriasis was evaluated simultaneously. The patient's psoriasis progressed, and the PASI score increased from 14.5 to 18 points over 2 weeks, during which skin biopsy was performed and a pathological diagnosis was made. The patient did not take any other medication during Histobulin™ therapy. The clinical response to Histobulin™ therapy was rapid, and the patient's symptoms and signs improved after the first injection of Histobulin™ (Figure 2). Although the patient temporarily showed some aggravation after the third injection, the clinical manifestations, including skin lesions, improved continually and completely disappeared after the eighth injection. The patient showed no symptoms or signs of psoriasis for 4 weeks, during which time 4 subsequent injections were administered. Treatment with the medication was stopped, and the patient did not experience recurrence for more than 18 months.

## 3 DISCUSSION

Psoriasis is a common dermatological disease.^13^ Moreover, other systemic diseases, including rheumatic disease, arthritis, colitis, diabetes, and hypertension, are frequently associated with psoriasis.^14^ Nevertheless, there is currently no cure for psoriasis, and conventional treatments are symptomatic.^15^ Many biologics have been developed and suggested for the treatment of psoriasis.^16^, ^17^ These biologics have their own associated side effects.^15^ Histobulin™ is a biologic therapeutic similar to IVIG.^18^ Histobulin™ has been used for several decades with considerable safety and without serious side effects. Moreover, Histobulin™ is not expensive, especially compared with other biologics.

Histobulin™ was effective in treating the psoriasis patient presented in this report. Moreover, the clinical response was very rapid, and complete remission was induced and maintained (Figure 2). This was the first episode of psoriasis for the patient. Regarding the clinical progression of psoriasis, the pathological findings indicated that the disease was in an early stage, and the clinical severity was mild to moderate. This case report involves early intervention in mild to moderately severe psoriasis. The rapid improvement seemed to be possible due to the mild to moderate severity of psoriasis in this patient. Notably, systemic inflammation accompanies psoriasis, and recently, early systemic treatment was recommended not only to improve cutaneous symptoms but also to reduce systemic inflammation, improving long‐term outcomes by mitigating comorbidity progression.^19^ This case report in which Histobulin™ was used describes early and systemic intervention for psoriasis. Early intervention seemed to be very effective in this patient.

Histobulin™ is an anti‐allergic therapeutic agent. A relationship between allergies and psoriasis has been reported. Immunoallergic reactivity has been reported in patients with psoriasis.^20^ In particular, the relationship between psoriasis and AD has been reported. Although psoriasis and AD are clearly independent diseases according to clinical criteria,^21^, ^22^ psoriasis and AD share a common immunopathogenesis.^23^ AD and psoriasis have even been suspected to be part of one spectrum.^24^ Recently, Histobulin™ was reported to be an effective treatment and to induce complete remission in AD patients.^11^ Considering the common immunopathogenesis of psoriasis and AD, Histobulin™ is naturally hypothesized to be effective in the treatment of psoriasis. However, with only one case, the relationship between allergic rhinitis and psoriasis could not be determined.

The intake of histamine‐rich foods was reported to lead to the development of various disorders in many organs with dermatological sequelae, including psoriasis.^25^ In psoriasis, tryptase‐ and chymase‐positive mast cells were activated early in developing lesions, and the cells later increased in number in the upper dermis with concomitant expression of cytokines and TNF superfamily ligands.^26^ Antihistamines for histamine receptor (HR) 1 were suggested as a treatment option for the symptom of itching in psoriasis.^27^ HR 2 antagonists were reported to have a clinical effect on psoriasis.^28^ In addition, histamine receptor 4 (HR 4) was reported to play a role in psoriasis.^29^ An action of histamine in the development of psoriasis has been suggested.^28^, ^30^ Histaminopexy is the main anti‐allergic mechanism of Histobulin™.^7^ Considering that histamine participates in the pathogenesis of psoriasis, Histobulin™ may be effective in the treatment of psoriasis.

Psoriasis is a chronic inflammatory autoimmune disease characterized by the excessive aberrant hyperproliferation of keratinocytes.^15^, ^31^ The pathogenesis of psoriasis is complex, and a strong proinflammatory stimulus leads to chronic inflammation in psoriasis patients.^15^, ^32^ Histaglobulin (the same immunoglobulin/histamine complex as Histobulin™) inhibits NF‐kappa B nuclear translocation and downregulates proinflammatory cytokines.^33^ The anti‐inflammatory effects of Histobulin™ were described in AD patients and patients with Pfeifer‐Weber‐Christian disease.^11^, ^34^ Histobulin™ may be effective in the treatment of psoriasis through anti‐inflammatory effects.

Intravenous immune globulin (IVIG) is well known to be effective in the treatment of autoimmune diseases,^35^ and IVIG is effective in the treatment of psoriasis patients.^4^, ^36^ The major constituent of Histobulin™ is immunoglobulin, and Histobulin™ may contain a small amount of IVIG. Histobulin™ may have effects on autoimmune disease. Recently, Histobulin™ was suspected to be effective in the treatment of patients with autoimmune diseases.^34^ The anti‐autoimmune effects of Histobulin™ possibly improved and induced complete remission in psoriasis in the patient presented in this case report. The mechanisms of action of Histobulin™ in psoriasis are listed as histaminopexic, anti‐inflammatory, and anti‐autoimmune effects as described above. The histaminopexic and anti‐inflammatory effects may be symptomatic and temporarily lead to the rapid improvement of clinical manifestations. However, the anti‐autoimmune effects may be causative, which may have induced complete remission without recurrence in the patient presented in this case report.

Conclusively, Histobulin™ is effective and induces remission through early intervention in patients with mild to moderate psoriasis. Curative treatments for psoriasis are lacking, and the development of safe and efficacious novel therapeutics is urgently needed for the treatment of psoriasis. Histobulin™ is suggested as a safe and inexpensive therapeutic with considerable clinical effects, as indicated in this report. Histobulin™ is also suggested as a curative therapeutic for psoriasis patients, and further basic research and clinical evaluation of Histobulin™ are necessary.

## AUTHOR CONTRIBUTIONS

GN performed the work for the clinical aspects of this report. HSK performed the work for the pathogenesis evaluation.

## CONFLICT OF INTEREST

The authors declare that they have no competing interests.

## ETHICAL APPROVAL

This case was approved by the IRB of Cheju Halla General Hospital (IRB No 2020‐M07‐01).

## CONSENT

Written informed consent was obtained from the patient for the publication of this case report and any accompanying images. A copy of the written consent is available for review by the editor of this journal.

## Data Availability

There are no conflicts of interest for the authors.
